# Relationship between Periodontal Diseases and Preterm Birth: Recent Epidemiological and Biological Data

**DOI:** 10.1155/2011/164654

**Published:** 2011-10-30

**Authors:** O. Huck, H. Tenenbaum, J.-L. Davideau

**Affiliations:** Department of Periodontology, Dental Faculty, University of Strasbourg, 67000 Strasbourg, France

## Abstract

For ten years, the incidence of preterm birth does not decrease in developed countries despite the promotion of public health programs. Many risk factors have been identified including ethnicity, age, tobacco, and infection. However, almost 50% of preterm birth causes remain unknown. The periodontal diseases are highly prevalent inflammatory and infectious diseases of tooth supporting tissues leading to an oral disability. They influence negatively general health worsening cardiovascular diseases and diabetes. Periodontal diseases have been also suspected to increase the rate of preterm birth, but data remain contradictory. The objective of this review is to present the principal results of epidemiological, biological, and interventional studies on the link between periodontal diseases and preterm birth. The conclusions of this work underline the importance for the physician/obstetrician to identify women at risk for preterm birth and to address these patients to dentist for periodontal examination and treatment in order to limit adverse pregnancy outcomes.

## 1. Introduction

Preterm birth is defined as any delivery that occurs after 23 gestational weeks and less than 37 weeks [1, 2]. This is a major determinant of neonatal morbidity and mortality [3]. Furthermore, preterm birth has long-term consequences for infant including an increased risk of neurological impairments and behavior disorders and higher rates of chronic health disorders than children born at term [4]. Global incidence of preterm birth is around 9.6% of all birth representing 12.9 million births [5] with regional disparities: 12% to 13% in the USA, from 5% to 9% in Europe [6], and 18% in Africa [7]. For ten years, the rate of preterm birth does not decrease in most of the industrialized countries. In the USA preterm birth prevalence increased from 9.5% in 1981 to 12.7% in 2005. Furthermore, women classified as black, Afro-American and Afro-Caribbean, are frequently reported to be at higher risk of preterm birth [7]. Preterm birth rates are in the range of 16–18% in black women compared with 5–9% for white women in USA [2]. Many preventive treatments have been proposed to decrease risk of preterm birth especially for women at risk. Many countries have programs offering special assistance to these women including advice and counseling (about nutrition, drugs, tobacco), assistance (transportation to clinic appointments, household help), and emotional support [8]. Obstetric treatments are possible including treatment with tocolytic agents, antenatal corticosteroids and antibiotics, and optimum timing of indicated preterm birth. These measures are intended to reduce the burden of prematurity-related illness more than to reduce the rate of preterm birth and have effects on perinatal morbidity [9]. 

The role of many risk factors have been shown by results of epidemiological studies such as increasing age of women giving birth, ethnical origin, tobacco, socioeconomic disparities, maternal body-mass index, or multiple pregnancies [5, 10–12]. Mother's health is also an important factor influencing pregnancy outcome. Cervical incompetence or short cervical length, preeclampsia and numerous maternal infection, systemic like toxoplasmosis [13, 14] and local infections such as bacterial vaginosis, chorioamnionitis, or uterine track infections [2, 15–17] increase the risk of preterm birth. Unfortunately, around 50% of causes of preterm birth remain unknown [16]. In 1996, Offenbacher et al. introduced the hypothesis that periodontal diseases could be a potential risk factor for preterm birth [18]. Since, many epidemiological or interventional studies have been performed to explore this relationship.

## 2. Periodontal Health and Pregnancy: A Reciprocal Relationship

The periodontal diseases are inflammatory diseases of gum and tooth supporting tissues caused by the oral bacterial biofilm containing almost 300 different species [19–21]. Different forms of periodontal diseases are observed. The superficial, reversible, and relatively harmless form corresponds to the gingivitis, and the profound and irreversible form corresponds to the periodontitis [20]. 

Gingivitis is a common pathology that affects everyone in his life (prevalence 80 to 100%). It corresponds to an inflammation of superficial soft tissues around teeth initiated by supragingival biofilms accumulation. The principal clinical signs are bleeding during tooth brushing or mastication, gum swelling, and gingival pains. Gingivitis is due to an absence or inappropriate oral hygiene habits and is worsened by local factor increasing dental plaque retention including supra-gingival calculus, retentive crown or dental misalignment, and absence or irregular dental cares. Consequently, oral hygiene education, scaling, and monitoring are very efficient to treat and prevent gingivitis [22]. 

**Figure 1:**
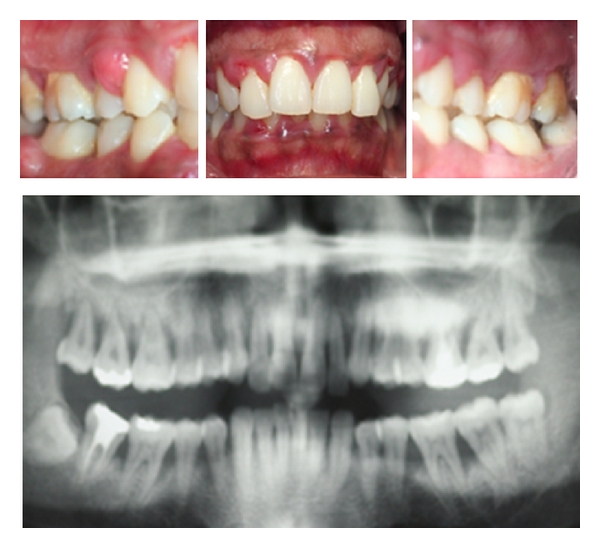
Clinical views of aggressive periodontitis affecting pregnant women. Major clinical signs are gingival inflammation and alveolar bone destruction. *(Courtesy to Dr. Bouaziz W)*.

The prevalence of periodontitis is about 60% with a pick of incidence at 60 years [20]. Periodontitis correspond to an inflammation of superficial and profound periodontal tissues caused by supra- and sub-gingival biofilms and leading to an irreversible destruction of tooth supporting tissues. The pathognomonic clinical sign of periodontitis is the formation of periodontal pockets. The others classical associated signs are gingival bleeding, gingival retraction, long appearance of teeth, tooth mobility, halitosis, abscess, bone loss, tooth mobility, and in the most severe cases, spontaneous tooth loss [20]. Two principal forms of periodontitis are recognized. The chronic form progresses slowly on many decades and displays successive phases of activity. Older patients may present severe form of chronic periodontitis with a consequent bone loss. However, in patients suffering from chronic periodontitis, the number of teeth lost for periodontal reasons is generally low. The aggressive form affects less than 10% of the general population. This form is predominant in young population and could lead to an important loss of teeth in few years [23]. This form is characterized by a strong inflammatory response and a rapid, profound, and generalized destruction of periodontal tissues associated to the presence of virulent bacteria such as *Porphyromonas gingivalis (Pg)*, *Aggregatibacter actinomycetemcomitans (Aa)*, and *Treponema denticola *[21] (Figure 1). Finally, the evolutions of chronic and aggressive periodontitis and at a lesser extent the evolution of gingivitis are markedly and negatively influenced by many risk factors such as tobacco, diabetes, low socioeconomic status, and ethnic origin. For instance, Africans have a higher prevalence of aggressive forms of periodontitis [24–26]. 

The diagnosis of gingivitis and periodontitis is generally based on clinical symptoms described above; however, be recognized by patient himself. Many studies based on self-report have shown that patients were able to evaluate correctly but grossly their periodontal status [27]. However, the evaluation of periodontitis severity requires a specific examination based at least on a periodontal probing performed by a periodontist or a general dentist. The periodontal pocket depth associated to gingival bleeding measurement could be considered as the best markers of periodontal disease activity or the inflammatory/infectious burden of periodontitis. The measurement of clinical attachment level (periodontal pocket depth plus gingival recession) and bone loss around teeth reflect more the history and the severity of periodontal disease. 

The aim of periodontitis treatment is to reduce periodontal tissues infection through a rigorous oral hygiene education and a mechanic treatment (scaling, root planning, and surgery) [20], associated to a chemical antimicrobial therapy including the administration of systemic antibiotics in the severe chronic or aggressive forms of periodontitis [28]. Patients must also attend regular visits to dentist or periodontologist to control and maintain periodontal treatment results. These treatments decrease efficiently periodontal tissue inflammation and eliminate the more virulent periodontal pathogens. They stop the destruction of periodontal tissues and prevent tooth loss [29–31]. Furthermore, they improve some systemic conditions (glycemia, lipid metabolism, endothelial function) [32–35]. However, the initial severity of periodontitis and the persistence of risk factors such as smoking and diabetes considerably impair periodontal treatment results [31, 36]. 

Interestingly, pregnancy influenced also periodontal status. Pregnant women are more susceptible to inflammation and display an increase of gingival bleeding on probing. Pregnant women present generally periodontal pocket due to gingival swelling rather than periodontal tissue breakdown [37]. These periodontal pockets disappear after delivery. However, in women suffering from periodontitis before their pregnancy, it appears that pregnancy could lead to an increase of periodontal disease severity [38]. Hormonal modifications have been proposed to exacerbate gingival inflammation, to initiate changes in the composition of oral biofilm, and to induce a selective growth of periodontal pathogens such as *Porphyromonas gingivalis*, *Prevotella intermedia *[39], or *Campylobacter rectus *[40].

## 3. Epidemiological Link

During the last decade, numerous epidemiological studies have been conducted on the association between preterm birth and periodontitis [3]. More or less strong associations between periodontal status and preterm birth alone (PB), low birth weight (LBW), or preterm birth associated to low birth weight (PLBW) have been shown in cohort/cross-sectional studies by Lunardelli and Peres [41] (Brazil, PB *P* < 0.02), Offenbacher et al. [42] (USA, PB *P* = 0.013), López et al. [43] (Chile, PLBW *P* < 0.0004 and RR = 3.5), Siqueira et al. [44] (Brazil, PB *P* < 0.001), Rajapakse et al. [45] (Sri Lanka, PB OR = 2.3), Toygar et al. [46] (Turkey, PB and PLBW *P* < 0.01), Agueda et al. [47] (Spain, PB OR = 1.77), and Heimonen et al. [48] (Finland, PB *P* < 0.001), and also in case-control studies by Gomes-Filho et al. [49] (Brazil, PLBW OR = 2.1), and Khader et al. [50] (Jordan, PLBW *P* < 0.0001). However, some other investigations did not find a significant association, such as cohort studies by Moore et al. [51] (PB, LBW), Noack et al. [52] (Germany, PLBW), Agueda et al. [47] (PLBW), Nabet et al. [53] (France, PB), and case-control studies by Davenport et al. [54] (UK, PLBW), Bassani et al. [55] (Brazil, PLBW), and Vettore et al. [56] (Brazil, PB, and PLBW). The different conclusions of these studies could be explained by the use of different definitions of adverse pregnancy outcomes, for instance PB versus PLBW and periodontal disease definitions, reflecting in fact different pathologic entities and disease severities [57, 58]. Indeed, the periodontal status assessment of pregnant women is mainly based on threshold numbers of sites with prespecified values of periodontal pocket depth and/or clinical attachment loss [41, 43, 47, 49, 59, 60] but could also be determined by the use of other composite index such as Community Periodontal Index for Treatment Need (CPITN) [48, 54] or other clinical signs including bleeding on probing [45]. Interestingly, the use of variable periodontitis definitions could reverse the association in some cases, especially in cohort studies [56, 58]. However, a high prevalence of severe periodontitis is frequently associated with PB and/or LBW [44, 47, 49, 60, 61] while a low prevalence (7.2%) is not [51]. Furthermore, the strength of the association between periodontal disease and preterm birth incidence increases frequently with the severity of periodontitis [7, 42, 49, 55, 58]. All these data suggest that women populations with a high prevalence of severe periodontitis are at risk for preterm birth. 

The risk factors of preterm birth appear to be similar to risk factors for periodontal diseases (tobacco, ethnicity, socioeconomic and educational levels) and may confound the association between periodontitis and preterm birth [3, 7, 62, 63]. Actually, smoking is recognized as one of the principal risk factors for both adverse pregnancy outcomes and periodontitis [17, 64]. In many studies evaluating periodontal status effect on pregnancy, the rate of smoking among pregnant women oscillates between 10 and 20% [41, 42, 46, 49, 55, 56] and is frequently related to periodontitis severity and/or preterm birth but not systematically. The definition criterion of smoking habits and severity, such as the number of cigarettes per day and period of smoking (during and/or prior to pregnancy), vary greatly between studies and may also explain these different results [46, 61]. In some studies ethnicity is correlated with pregnancy outcome and/or periodontal status [42, 51, 63] while other investigations do not report such a correlation [54–56, 65]. In the same way, elevated percentages of pregnant women with no education or only primary education are frequently associated with PB and/or LBW [44, 46, 50] and periodontitis [45], but not systematically [54, 55]. This diversity of epidemiologic study results shows the interest to best define the specificity of periodontal pathology of pregnant women considering their young age and the hormonal influence of pregnancy on periodontal tissues.

## 4. Biological Hypothesis

Considering epidemiological evidence, biological theories have been proposed to link preterm birth and periodontal diseases [66]. Mainly, three hypotheses are developed

- bacterial spreading,
- inflammatory products dissemination,
- role of feto-maternal immune response against oral pathogens.

### 4.1. Bacterial Spreading

The current paradigm indicates that majority of intrauterine infection originates in the lower genital tract [67]. Despite this statement, number of studies report intrauterine infections caused by species not found in urogenital tract [67]. The bacterial spreading theory is based on the possible dissemination of oral bacteria including periodontal pathogens through blood circulation [68] to the amniotic fluid and leading to chorioamniotic infections. The frequent gingival inflammation of women presenting periodontal diseases [69], especially pregnancy-associated gingivitis [70], facilitates bacteremia process [67]. Furthermore, the more periodontal pockets are deep, the more important is the exchange surface between bacteria biofilm and blood circulation (15 to 20 cm^2^ in the most severe cases) [71]. Many analyses of amniotic fluid or placenta have been performed and evidence the presence of different oral pathogens such as *Bergeyella*, *Eikenella *[67], *Fusobacterium nucleatum, *or *Porphyromonas gingivalis *[72–74]. Inside uterus, these pathogens could provoke an inflammatory response. The increase of inflammatory cytokines or metalloproteases synthesis and the neutrophil activation could induce preterm birth process [67]. 

*In vivo *studies show that the invasiveness of uterine tissues largely depends on the type of bacteria. In a sheep model of intra-amniotic injection of lipopolysaccharide from different bacterial species, it appears that periodontopathic lipopolysaccharides induce a high rate of fetal lethality [75]. Furthermore, in a rat model, placenta colonization by *Porphyromonas gingivalis *is dose and strain dependent [76]. Potential pathological mechanisms of certain periopathogens, especially for *Porphyromonas gingivalis *and *Fusobacterium nucleatum*, have been studied. For example, *Porphyromonas gingivalis *could infect syncytiotrophoblasts, chorionic trophoblasts, decidual cells, and amniotic epithelial cells [74] and promotes inflammatory process trough Toll-like receptor 4 [77]. 

Finally, a case-report study has been published in 2010 concerning a stillbirth caused by *Fusobacterium nucleatum *from the mother's mouth [78]. This study highlights the fact that an oral periodontal pathogen can, by hematologic pathway, colonize placenta and provoke fetal complications. It is important to notice that such colonization may be dependant from mother's immunological status.

### 4.2. Hematogenous Dissemination of Inflammatory Products

Acute inflammation is responsible for a substantial fraction of preterm birth [79]. In 1998, Offenbacher et al. suggested that the cytokines produced by local inflammation in periodontal tissues affected by periodontitis have systemic effects after diffusion of such cytokines through blood flow [80]. Locally, studies show that periodontal diseases increase secretion of several cytokines, notably PGE-2, TNF-*α*, IL-6 or IL-1*β* [81, 82]. Analysis of amniotic fluid obtained at the time of preterm birth shows elevated levels of inflammatory cytokines [83]. We hypothesize that cytokines produced in periodontal tissues promote inflammation in maternal-fetal unit. Clinically, high gingival crevicular fluid levels of PGE-2, IL-1*β*, or Il-6 have been associated with their elevated levels in amniotic fluid [80, 84]. The inflammatory response appears to be the privileged pathway of the pathogenic periodontal disease influence on pregnancy, as suggested for other major systemic diseases including cardiovascular diseases or diabetes [85].

### 4.3. Fetomaternal Immune Response

The immune and genetic characteristics of fetus and pregnant women are one of the potential mechanisms linking periodontal diseases to preterm birth. Numerous studies have analyzed fetal and maternal antibodies directed against oral pathogens during pregnancy. In the study of Boggess et al., 35.2% of samples are Ig-M positive for at least one oral pathogen, and 26.6% are positive for more than one. The presence of Ig-M is associated to an increased risk of preterm birth [86]. This immune response against oral pathogens could be associated with an inflammatory response, and the synergy between the two mechanisms increases significantly the risk [86]. The genetic predisposition is also important. Polymorphisms of genes coding for proinflammatory cytokines such as TNF-*α*, IL-1 or IL-6 are associated to a hyperinflammatory response. The consecutive overexpression of these cytokines increases the risk of preterm birth [87–89]. 

The mechanisms linking periodontal diseases and preterm birth are not well defined. Further investigations should be performed to evaluate the impact of each theory. Nevertheless, we hypothesized that the influence of periodontal diseases on preterm birth is the result of an inflammation of the fetomaternal unit that is amplified in women presenting particular phenotype.

## 5. Effects of Periodontal Treatment on Preterm Birth Incidence

Considering periodontal diseases as a risk factor for preterm birth, interventional studies have been performed to evaluate impact of periodontal treatment on pregnancy outcomes. Case-control studies including a relative large number of pregnant women (>400) show some apparent contradictory results and different conclusions [43, 90–94]. Indeed, the periodontal treatment may improve periodontal conditions and/or pregnancy outcomes [43, 90, 94] or not [91, 93]. A recent meta-analysis indicates that the treatment of periodontal diseases does not reduce the rate of preterm birth [95]. However, as discussed above for epidemiological studies, the conclusions of this analysis could be balanced by the relative heterogeneity of studied populations, according to risk factors ethnicity, smoking, socioeducative levels, and periodontal status definition. For instance, the percentage of black people varies considerably between studies: 50% to 65% of Hispanic and Caucasian [38, 43]); 45% to 87% of Afro-American [90, 93]. Furthermore, the modalities of periodontal care in the different studies display some differences that may influence periodontal outcomes. A first session of etiologic periodontal treatment, including oral hygiene instructions, scaling, and root planning was generally performed at the end of the first trimester of pregnancy (before 20 to 28 weeks). This first session could be unique [91, 93] or reinforced by regular control visits and complementary treatments if necessary until delivery [43, 90]. The local effects of periodontal treatments are generally positive [43, 90]. Gingival inflammation and mean probing pocket depth are reduced, especially in study using reinforced periodontal treatment modalities [43, 90]. However, a relative high rate of patients demonstrating a periodontitis progression is observed in some studies (70% [91], 50% [90], 68% [94]) suggesting that periodontal treatments do not work so efficiently than in a general population [91]. Indeed, the relative “narrow therapeutic window” to perform periodontal treatment and to obtain a successful periodontal lesion cicatrisation, and the aggressive profile of severe periodontitis in young women could be considered as limiting factors [91, 94]. A recent study performed by Jeffcoat et al. [94] confirms that the efficiency of periodontal treatment should be considered before the analysis of results. In this study, 322 pregnant women with periodontal disease have been followed, 160 have received randomly complete periodontal treatment, and 162 have served as control without treatment. No significant difference was found in term of preterm birth incidence between the two groups. However, after considering the effect of periodontal therapy, the results demonstrate a strong and significant relationship between successful periodontal treatment and full-term birth ratio (odds ratio = 6.02) [94]. 

Despite apparent conflicting data, the majority of studies report that periodontal treatment is safe for pregnant women and improve periodontal status [3, 90, 92, 94]. The pregnant woman is a particular patient. In order to decrease the impact of periodontal disease on preterm birth incidence, the early diagnosis promotion of periodontal disease for young women especially for those presenting major risk factors should be recommended. The preventive oral care is the best way to prevent oral diseases and their consequences on pregnancy [96]. In case of periodontal disease diagnosis done during pregnancy, a frequent monitoring of the patient should be positive for the control of the disease and the decrease of preterm birth risk. For severe or aggressive periodontitis, metronidazole or amoxicillin could be used in addition to mechanical treatments. Studies have been performed on the effect of antibiotic on rate of preterm birth concerning bacterial vaginosis. The majority of them demonstrates no deleterious effect of antibiotic use on pregnancy outcome especially for metronidazole [97].

## 6. Conclusion

Periodontal diseases appear to be a potential risk factor for preterm birth. As well as other modifiable risk factors, these diseases must be taken in charge. Cooperation between obstetricians or general practitioners and periodontists should be developed. The promotion of the early detection and treatments of periodontal disease in young women before and during pregnancy will be beneficial especially for women at risk.
